# Digital Contact Tracing Based on a Graph Database Algorithm for Emergency Management During the COVID-19 Epidemic: Case Study

**DOI:** 10.2196/26836

**Published:** 2021-01-22

**Authors:** Zijun Mao, Hong Yao, Qi Zou, Weiting Zhang, Ying Dong

**Affiliations:** 1 College of Public Administration Huazhong University of Science and Technology Wuhan China; 2 Non-traditional Security Research Center Huazhong University of Science and Technology Wuhan China; 3 School of Law and Humanities China University of Mining and Technology Beijing China

**Keywords:** COVID-19, digital contact tracing, emergency management, graph database, big data, visualization, China

## Abstract

**Background:**

The COVID-19 epidemic is still spreading globally. Contact tracing is a vital strategy in epidemic emergency management; however, traditional contact tracing faces many limitations in practice. The application of digital technology provides an opportunity for local governments to trace the contacts of individuals with COVID-19 more comprehensively, efficiently, and precisely.

**Objective:**

Our research aimed to provide new solutions to overcome the limitations of traditional contact tracing by introducing the organizational process, technical process, and main achievements of digital contact tracing in Hainan Province.

**Methods:**

A graph database algorithm, which can efficiently process complex relational networks, was applied in Hainan Province; this algorithm relies on a governmental big data platform to analyze multisource COVID-19 epidemic data and build networks of relationships among high-risk infected individuals, the general population, vehicles, and public places to identify and trace contacts. We summarized the organizational and technical process of digital contact tracing in Hainan Province based on interviews and data analyses.

**Results:**

An integrated emergency management command system and a multi-agency coordination mechanism were formed during the emergency management of the COVID-19 epidemic in Hainan Province. The collection, storage, analysis, and application of multisource epidemic data were realized based on the government’s big data platform using a centralized model. The graph database algorithm is compatible with this platform and can analyze multisource and heterogeneous big data related to the epidemic. These practices were used to quickly and accurately identify and trace 10,871 contacts among hundreds of thousands of epidemic data records; 378 closest contacts and a number of public places with high risk of infection were identified. A confirmed patient was found after quarantine measures were implemented by all contacts.

**Conclusions:**

During the emergency management of the COVID-19 epidemic, Hainan Province used a graph database algorithm to trace contacts in a centralized model, which can identify infected individuals and high-risk public places more quickly and accurately. This practice can provide support to government agencies to implement precise, agile, and evidence-based emergency management measures and improve the responsiveness of the public health emergency response system. Strengthening data security, improving tracing accuracy, enabling intelligent data collection, and improving data-sharing mechanisms and technologies are directions for optimizing digital contact tracing.

## Introduction

The COVID-19 epidemic is still spreading rapidly worldwide. Tens of millions of people have been infected, and the number of infections is still growing rapidly. Most countries or regions are still in states of public health emergency. Although China, the United States, and other countries have successfully developed COVID-19 vaccines, the production and application of the vaccines still have large gaps. Moreover, effective drugs for treatment of COVID-19 have not been successfully developed. Therefore, quickly identifying and tracing individuals infected with COVID-19 and their contacts and adopting active emergency management measures, such as travel restrictions, health monitoring, and home-based quarantine, are necessary for all countries and regions to overcome the COVID-19 epidemic [1].

Contact investigation has always been an important public health strategy and a key process in epidemic emergency management. The scale of COVID-19 infection poses a major challenge to contact investigation [2]. Traditional manual contact investigation is conducted through telephone or face-to-face interviews, and these processes are usually slow, laborious, and inefficient; therefore, it is difficult for health officials and disease control staff to obtain all the information of all contacts [2-5]. In addition, traditional manual contact tracing is not effective in identifying and managing asymptomatic and mildly symptomatic infected individuals, who account for 84% of the total number of infections [6,7]. Traditional manual contact tracing is also unlikely to be implemented for displaced migrants [8]. Some medical experts believe that COVID-19 may develop into a seasonal epidemic, similar to influenza, which creates great challenges for traditional manual contact tracing [9].

Digital contact tracing uses electronic information to identify exposures to infection; it has the potential to address the limitations of traditional contact tracing, such as scalability, notification delays, recall errors, and contact identification in public spaces [10,11]. Current digital technology enables the continuous tracing of individuals and locations using mobile phones, GPS, wireless fidelity (Wi-Fi), and Bluetooth to record and trace the spatiotemporal trajectory of individuals to identify potential contacts [12,13]. These systems can help overcome the limitations of manual contact tracing [12]. An empirical study proved that the widespread use of digital contact tracing can reduce the spread of epidemics, and far fewer individuals are placed in isolation when such systems are applied [12].

The data storage and processing models of digital contact tracing adopted by some countries and regions are generally divided into centralized and decentralized models [14]. In the centralized model, anonymized data are uploaded from people’s contact information to centralized servers; then, health authorities can check, notify, and manage previous contacts [14,15]. In comparison, the decentralized model locally stores these key codes and allows users to (1) notify the system if they have tested positive (or not), so the mobile app will upload the last 14 days of locally stored keys to the server; and (2) voluntarily check their risk exposure, that is, whether they have been in contact with someone who may have been infected, by downloading the uploaded keys from the server and matching locally against the stored keys to evaluate their risk of exposure [14].

South Korea and China have adopted the centralized model for digital contact tracing [2]. Korea’s Center for Disease Control and Prevention (CDC) built the COVID-19 Smart Management System based on big data, such as security cameras, credit card records, and even the GPS of cars and mobile phones, to trace people who may be exposed to COVID-19 and to implement quarantine measures [16]. The mainland Chinese government has developed a smartphone applet to collect residents’ self-reported health characteristics and travel history and to combine the location information generated by the users’ specific quick response (QR) codes to trace infected persons and contacts for individual risk assessment. A health code is generated for the identification of personal infection risk; this code serves as a voucher for passage, work resumption, and school resumption [10,16]. The COVID-19 epidemic emergency management strategies in Hong Kong and Taiwan focus on the isolation of imported cases. Hong Kong has designed a compulsory electronic wristband with a mobile app, which all travelers entering Hong Kong are required to wear [16]. The device alerts the authorities when a traveler leaves their designated quarantine place [16]. Taiwan authorities used the National Health Insurance Database and the Immigration and Customs Database to match symptoms and travel experiences to identify people who may have been exposed to COVID-19 [16].

The decentralized model was adopted by countries and regions such as Europe, North America, and Singapore [2]. Google, Apple, the Massachusetts Institute of Technology, Singapore authorities, and some European consortia have developed apps based on smartphone Bluetooth technology that can notify users if they have been in contact with confirmed patients [2,10,17]. These apps use the Bluetooth signal strength to infer the distance between smartphones and define the user’s exposure status based on the distance and time of proximity to individuals who are subsequently identified as infected. The apps are adopted with users’ permission and do not collect user data in any centralized digital or information system [2,10,17].

App-based digital contact tracing in a decentralized mode can only be effective when used by 40%-70% of smartphone users [3]. The early results of the adoption of Singapore’s Bluetooth-based digital contact tracing app showed that only 20% of the population had installed the app as of April 21, 2020, because of its operational complexity, the limitations of the underlying technology, and citizen trust [2]. The proportions of countries in Europe and North America that adopted such apps were even lower [15]. Europe, North America, and other Western regions are relatively adverse to digital contact tracing under the centralized model due to considerations of public freedom and privacy; these countries also rejected strict emergency management measures for similar reasons at the beginning of the COVID-19 epidemic, which led to uncontrolled spread of the epidemic [18,19]. Fortunately, after recognizing the importance of strict emergency management measures, Western countries subsequently adopted measures to address the COVID-19 epidemic and achieved good results. Practices have proved that governments must take a more active role in COVID-19 epidemic emergency management; digital contact tracing in a centralized model may become the mainstream measure for major epidemic emergency management worldwide.

An empirical study [14] showed that the use of a centralized model for digital contact tracing is more effective, and the minimum utilization or coverage rate of the centralized model in epidemic control is remarkably lower than that of the decentralized model; however, this study ignored the unique problems faced by the centralized model in the implementation process. Taking Mainland China as a typical example, the health code applets developed in Mainland China based on WeChat social software and Alipay payment software have high utilization rates, which may meet the requirements for effective contact tracing. However, the objectivity of health codes is insufficient, and their effectiveness is weakened because of the autonomy of users to report their health characteristics and contact history and the arbitrariness with which users scan designated QR codes in various places to determine location information.

Therefore, this study aimed to overcome the insufficient use and objectivity of existing digital contact tracing–related practices and provide new solutions to further improve the effectiveness and reliability of digital contact tracing. Hainan Province, China, was selected as the case in this study. Hainan Province adopted a centralized model to conduct contact tracing during the COVID-19 epidemic. Moreover, the model gathered multisource epidemic data that relied on the government’s big data public service platform, which enabled government agencies to apply graph database algorithms, data visualization, and other digital technologies to determine and trace contacts from hundreds of thousands of epidemic records. This study describes the organizational process, technical process, application prospects and possible obstacles of digital contact tracing in Hainan Province, which may provide a more effective solution and technical support for other countries and regions.

## Methods

### Case Selection

Hainan Province is one of the most popular tourist destinations among Chinese and even global tourists because of its tropical island scenery. Due to the massive influx of tourists, the flow rate of the population of Hainan Province is very large. Additionally, due to the geographical location of the island, the influx of infected individuals is the main challenge in the emergency management of the COVID-19 epidemic. As of August 7, 2020, Hainan Province reported a total of 171 confirmed individuals infected with COVID-19 [20]. The vast majority of these cases were identified between January 22 and February 19, 2020 (168 cases); only 3 cases appeared in the next 6 months [20]. This outcome proved the success of the emergency management strategy of the COVID-19 epidemic in Hainan Province.

Hainan Province issued the “Regulations on the Development and Application of Big Data in Hainan Province” in October 2019 [21], which clarifies relevant regulations on the development and sharing of big data, application and industry promotion, data security, and legal responsibility at all levels of government agencies. According to the regulations, Hainan Province established the Big Data Administration and the big data public service platform to collect the transaction information system data of government agencies at all levels [21]. These regulations helped Hainan Province become one of the first provinces in China to use digital technology to respond to the COVID-19 epidemic [22]. Hainan Province relied on the government’s big data public service platform to gather epidemic multisource big data and used graph database algorithms to determine and trace contacts, which achieved good results. Hainan Province was selected as the case in our research to better demonstrate the innovative ideas and technologies of digital contact tracing development in the context of big data.

### Materials

#### Qualitative Materials

We conducted semistructured interviews in collaboration with the project manager and technical staff of the Big Data Administration who were in charge of Hainan’s COVID-19 epidemic digital contact tracing project to understand the details of the whole process. We explained the purpose and content of the interview to the interviewees before the interview, recorded the outlines during the interview, and transcribed the voice recording to text format after the interview.

#### Quantitative Materials

Under the leadership and coordination of the Command of Hainan Provincial Epidemic Prevention and Control, the Hainan Provincial Big Data Administration collected epidemic data from different agencies, including epidemic investigation records, confirmed infected individuals’ information and their spatiotemporal trajectory, high-risk population information, information on close contacts and patients with fever, and the mobile phone signaling data of imported residents or travelers with a history of residence in Hubei Province (patients with COVID-19 were first reported in Wuhan, Hubei Province, and Hubei Province accounted for the majority of cases in China). The information in the specific databases is shown in Table 1. The technical personnel organized by the Hainan Provincial Big Data Administration cleaned and integrated the multisource epidemic data, then uploaded the data to the government’s big data public management platform. The platform provided hive data warehouse tools to help authorized personnel manage, extract, query, and analyze data. The time range covered by all the databases was January 21 to February 9, 2020. The collection, storage, and analysis of all data in this study were completed by the administrative officials and technical personnel authorized by the Hainan Provincial Big Data Administration. The authors of this paper had no authority to participate in any specific process. The only information possessed by the authors was the organizational process, technical process, and analysis results provided by the Hainan Provincial Big Data Administration. No data or information in this study involved citizens’ sensitive or private information.

**Table 1:** Data collection list of COVID-19 epidemic digital contact tracing in Hainan Province.

Database	Data provider	Description	Sample size (n)	Collection method
Resident trajectory information database from Hubei Province	Health Committee	Used to understand where citizens have gone and who they have contacted	205,833	The health committee organizes the disease prevention and control center, community health service agencies, and community (village) resident committees to conduct household surveys and telephone verifications.
Confirmed Person Information Database	Public Security Department; Health Committee	Used to understand which patients have died or have recovered and been discharged	163	Reported by medical and health institutions
Information database of high-risk groups and close contacts	Epidemic Prevention and Control Headquarters	Used to understand the close contacts of high-risk individuals in confined spaces	2,269	Summary of Center for Disease Control information
Hospital fever information database	Epidemic Prevention and Control Headquarters	Used to understand who was infected, where the person was treated, whether the infected person recovered, and where the infected person lives	113,606	Hospitals report through the health committee information system
Mobile phone signaling database	Communications operator branch	Used to understand where people moved to and are staying	231,296	Directly provided by China Mobile, China Unicom, China Telecom, and China Broadcasting Network Corp, Ltd

### Main Methods

#### The Graph Database and Its Algorithm

A graph database is a new type of database system based on graph theory and algorithms that efficiently processes complex relational networks. A graph database can efficiently process large-scale, complex, interconnected, and changeable data, and its computational efficiency is far higher than that of a traditional relational database [23]. A graph database is scalable and flexible, is suitable for complex relationship management and relationship query reasoning, and reveals implicit relationships between entities [24].

**Figure 1:**
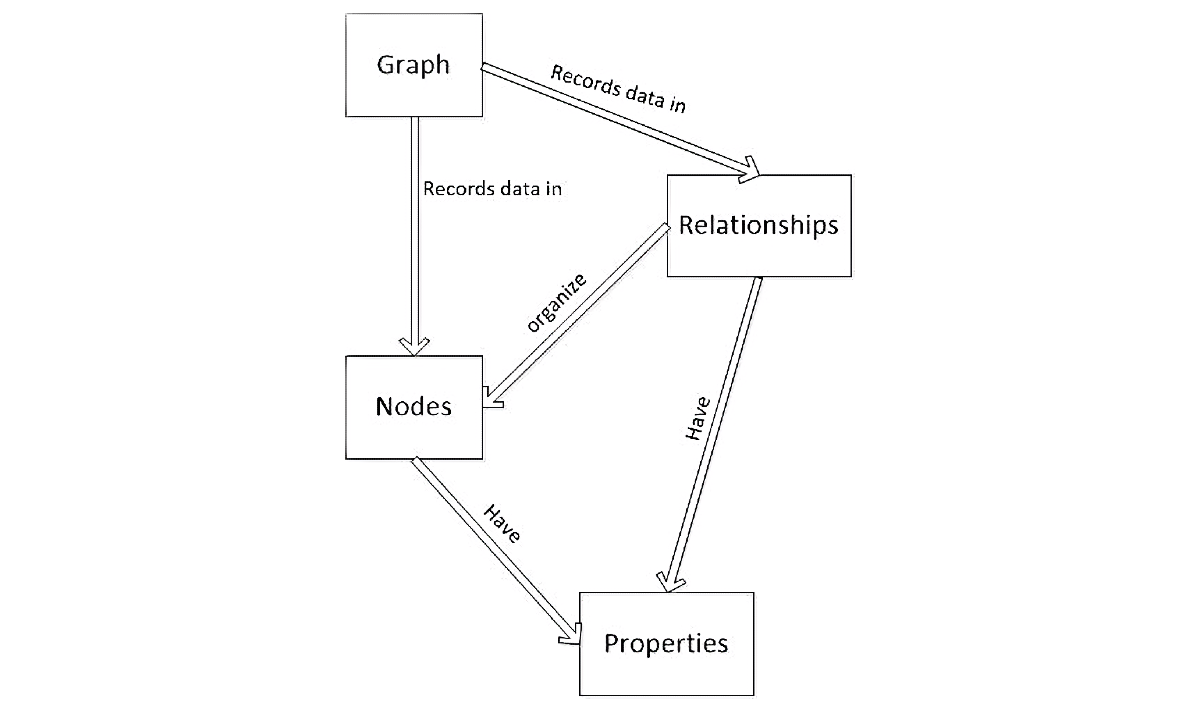
Graph database model based on Neo4j.

Most graph databases provide a query language that is suitable for representing graph structures and graph query. Neo4j (Neo4j, Inc) is a Java-based open-source graph database with high performance, high reliability, and strong scalability [23]. The graph database model based on Neo4j is shown in Figure 1. The information modeling of Neo4j includes three structural units: nodes, relationships, and properties [25]. Each node in the database graph can establish a relationship with any other node, and each node can set multiple properties [25]. Each relationship in the graph must have a start node and an end node, and each relationship can also set multiple properties [25].

Our research used the Cypher algorithm of Neo4j version 3.4.15 to construct an association graph among the high-risk population, the general population, vehicles, public places, and other key information; reveal the hidden network of relationships; and identify the risks in the relationships to identify and trace the contacts of COVID-19 cases.

#### Data Visualization and Interactive Operating System Based on ECharts

ECharts (Apache Software Foundation) is an open-source visualization tool based on JavaScript that can run smoothly on personal computers and mobile devices and is compatible with most current web browsers. The bottom layer relies on a vector graphics tool, ZRender, to provide intuitive, interactive, and highly personalized data visualization [26]. ECharts uses incremental rendering technology (4.0+) with various detailed optimizations to display tens of millions of data points, and it can still perform smooth zoom and pan interactions at this data level [26]. ECharts provides legends, visual mapping, data area zoom, tooltips, and data-filtering interactive components, which can perform multidimensional filtering and view zooming and display details [26].

Our research used ECharts to visualize basic data and the results of the graph database algorithms as well as to develop web portals and interactive operating systems to intuitively and dynamically query and analyze epidemic data.

## Results

### Organizational Process of Digital Contact Tracing During the COVID-19 Epidemic in Hainan Province

Based on the data planning, data collection, data storage, data analysis, and data application processes involved in the data life cycle, we used interview data to summarize the activities performed by various participants in the digital contact tracing process of the COVID-19 epidemic in Hainan Province. The specific organizational process is as follows.

#### Data Planning Process

Data planning includes organization, assessment of the situation and demand, and the formulation of strategic goals. This project was initiated by the Hainan Provincial Command of COVID-19 Epidemic Prevention and Control on January 29, 2020. The governor of Hainan Province, as the person in charge, instructed various government departments to cooperate with the Big Data Administration to apply big data technology to COVID-19 epidemic emergency management–related work. That is, the Hainan Provincial Command of COVID-19 Epidemic Prevention and Control played a leading and coordinating role, the Hainan Big Data Administration played a leading role in implementation, and other government departments played cooperative roles. Thus, an integrated and flat organizational structure and coordination mechanism were formed.

The Hainan Big Data Administration immediately assessed the COVID-19 epidemic situation and emergency management measures, coordinated communication with the Command of COVID-19 Epidemic Prevention and Control and other relevant government departments, and organized epidemiologists and big data technicians to formulate an implementation plan. The plan determined the specific implementation details of using multisource epidemic big data for digital technology tracing and submitted achievable data requirements to the Command of COVID-19 Epidemic Prevention and Control. Then, the COVID-19 Epidemic Prevention and Control headquarters coordinated the cooperation of work by relevant departments and provided the specified data.

#### Data Collection Process

Hainan Provincial Big Data Management collected the first batch of data in Excel format (Microsoft Corporation) from the Command of COVID-19 Epidemic Prevention and Control, Public Security Department, Health Commission, communication operators, and other departments on February 10, 2020.

#### Data Storage Process

The technicians of the Hainan Provincial Big Data Administration cleaned and merged the data under the guidance of officials and epidemiologists, uploaded all databases to Hainan’s big data public service platform, and used the hive data warehouse tool to manage, extract, query, and analyze the data.

#### Data Analysis Process

The data technicians of the Hainan Provincial Big Data Administration retrieved data from Hainan’s big data public service platform; used the graph database Neo4j algorithm to perform association analysis on key populations, vehicles, and public places; and then used ECharts to visualize the data analysis results and determine contacts and high-risk public places.

#### Data Application Process

The Hainan Provincial Big Data Administration regularly writes COVID-19 epidemic contact tracing reports based on the results of data analysis and reports them to the Command of COVID-19 Epidemic Prevention and Control. The Command of COVID-19 Epidemic Prevention and Control promptly releases early warning information to the public; strengthens cross-departmental sharing of information; and guides the Health Commission, CDC, medical and health institutions, and grassroots residents’ committees in the implementation of measures, such as isolation, health monitoring, and nucleic acid testing of contacts. Measures are also implemented to limit the flow of people and close high-risk infected public places.

### Technical Process of Digital Contact Tracing During the COVID-19 Epidemic in Hainan Province

#### Application of the Graph Database Algorithm to Determine and Trace Contacts

The technicians of the Hainan Provincial Big Data Administration took confirmed, suspected, and asymptomatic infected individuals, as well as individuals with a history of residence in Hubei Province, as the nodes of population; private cars, trains, and flights as the nodes of transportation; and communities and shopping malls as the nodes of public places. The technicians defined a relationship as appearance in the same public place or vehicle at the same time. Based on these nodes and relationships, the Neo4j Cypher algorithm graph database was applied to build an association graph to analyze and display the associations among key populations, vehicles, public places, and other key pieces of information. The core algorithm of the graph database used in our research is shown in Table 2.

**Table 2:** Hainan provincial digital contact tracing core algorithm based on a graph database.

Algorithm function	Specific algorithm	Algorithm description
Tracing the travel companions of confirmed, suspected, and asymptomatic infected individuals	MATCH p=(n:Individual)-[r:'sameTransportation']-(n1)-[rr:'sameTransportation']-(n2) where n.name<>n2.name and upper(n.ID) in ['ID1','ID2','ID3','ID4','ID...'] RETURN distinct n.name,upper(n.ID),n.phoneNo,n1.name,upper(n1.ID),n1.phoneNo,n2.name,upper(n2.ID),n2.phoneNo；	“Individual” means the individuals included in the analysis; “sameTransportation” means taking one mode of transportation at the same time; “Name” means the name of the analyzed objects; “ID” is the ID number, where “ID/ID1/ID2/ID3/ID4/ID...” are the ID numbers of confirmed, suspected and asymptomatic infected individuals;”phoneNo” is the mobile phone number.
Tracing individuals who have had contact with >2 confirmed infected individuals	MATCH p=(n:'Individual')-[r1]->()<-[r2]-(nm)-[r21]->()<-[r22]-(m:'Individual') where n.is_confirmedindividual in ['yes','sameID'] and m.is_confirmedindividual in ['yes','sameID'] and EXISTS(nm.is_confirmedindividual)=false and n.ID<>m.ID return p;	“confirmedindividual” means a confirmed individual; “sameID” means the same ID number.
Tracing contacts through transportation modes with high risk of infection	match p=(n:Transportation)-[*..4]-() where n.name='CarNo1' return p;	“Transportation” means the transportation included in the analysis (private car, train, airplane); “CarNo1” is the license plate number of a certain private car.

**Figure 2:**
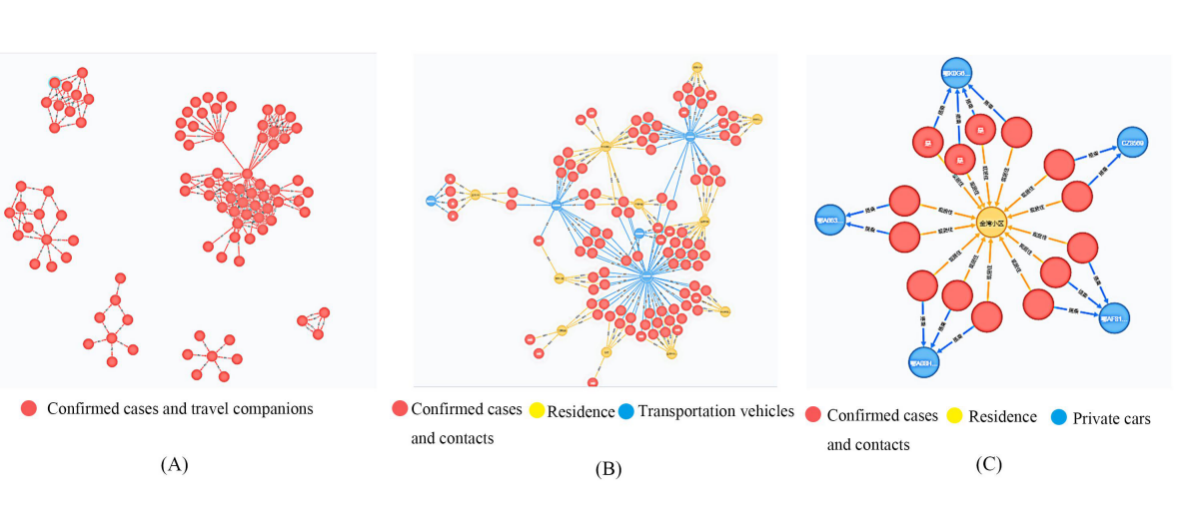
Contact tracing based on the algorithm of the Neo4j software package for the graph database: (A) contacts associated with travel, (B) contacts associated with >2 confirmed cases, and (C) contacts associated with private cars.

The representative analysis results of the graph database algorithm are shown in Figure 2. Figure 2A shows the close contacts of confirmed cases who rode in the same vehicle and the second-degree and higher-degree contacts associated with the abovementioned close contacts in other vehicles. Figure 2B shows the close, second-degree, and higher-degree contacts associated with >2 confirmed cases through any means of transportation or public place. Figure 2C shows the close contacts associated with confirmed cases through private cars and communities. The Neo4j software package supports the export of the information on the close, second-degree, and higher-degree contacts in Excel format so that the authorities can implement quarantine measures on the contacts.

#### Application of the Graph Database Algorithm to Determine Public Places With High Risk of Infection

**Figure 3:**
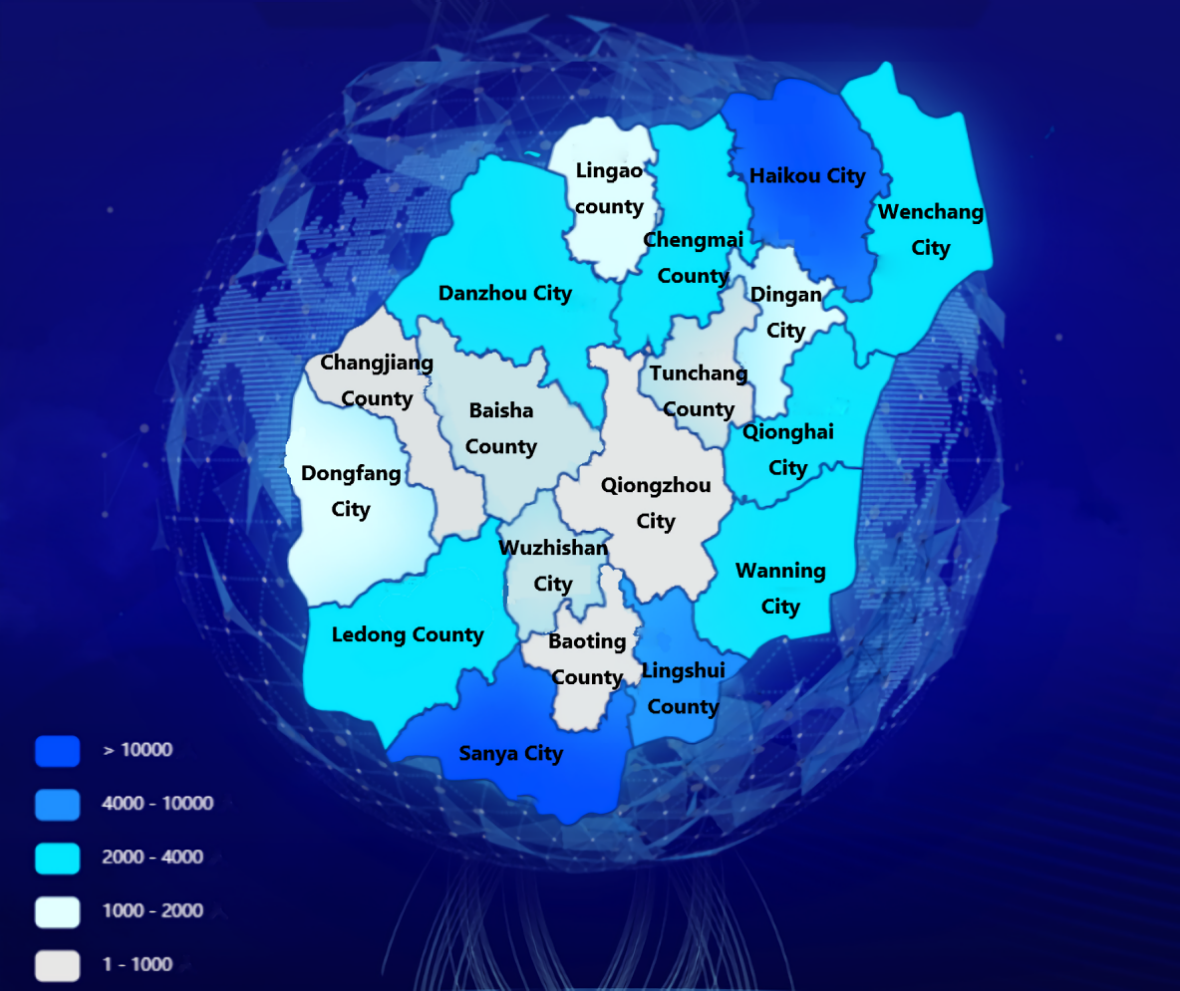
Distribution of the population of residents of Hainan Province who have a history of residence in Hubei Province.

The Hainan Provincial Big Data Administration applied ECharts data visualization tools to compute and visualize the distribution of the population with a residence history in Hubei Province in all cities of Hainan Province. As shown in Figure 3, Haikou, the administrative center of Hainan Province, and the well-known tourist city, Sanya, have the largest numbers of imported residents with a history of residence in Hubei Province; both populations were greater than 10,000. Overall, the population of individuals in Hainan Province with residence history in Hubei Province was more concentrated in coastal cities than in central cities.

The Hainan Provincial Big Data Administration applied the graph database algorithm to rank the frequency of confirmed, suspected, and asymptomatic infected individuals and their contacts appearing in shopping malls and communities. The results were used to infer and predict public places with high risk of infection.

#### Application of ECharts to Design Data Visualization and an Interactive Operating System

**Figure 4:**
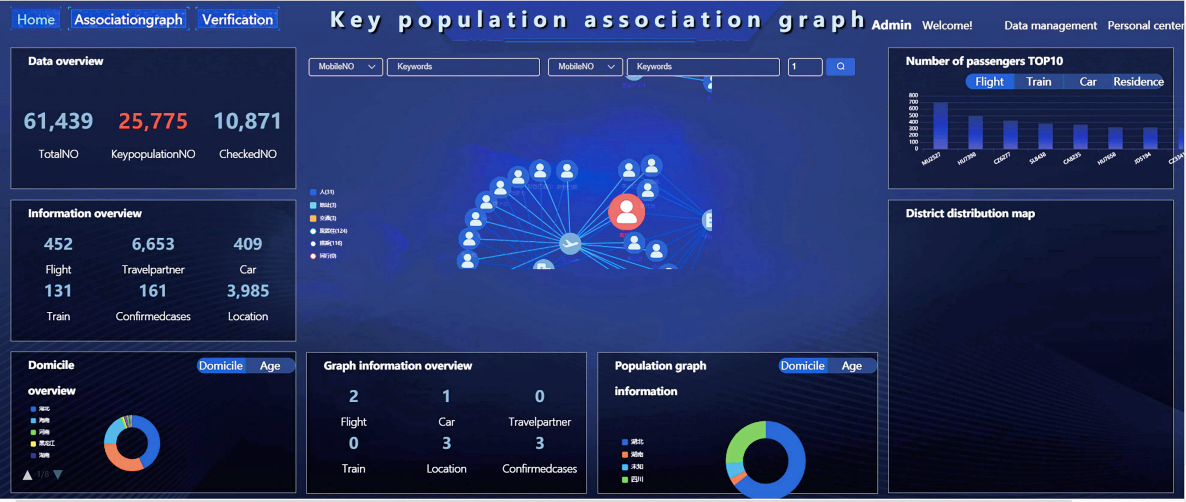
Data visualization and interactive system interface designed with ECharts.

The Hainan Provincial Big Data Administration used ECharts to visualize the basic data and analysis results and also designed an interactive operating system that can be accessed through a web page on a browser. The system interface is shown in Figure 4. The interactive operating system displays the total amount of data processed, the overview of epidemic prevention and control information, the statistics of the permanent residence of the imported persons from Hubei Province, the overview of the graph information, the information of the persons involved in the graph, and the distribution of high-risk infected individuals in transportation vehicles and public places. In addition, the system provides a query function of the graph analysis results so that administrative officials and disease prevention and control staff can directly view the graph results by keyword query.

### Main Achievements of Digital Contact Tracing During the COVID-19 Epidemic in Hainan Province

The Hainan Provincial Big Data Administration identified 61,439 analysis objects from hundreds of thousands of records and identified 10,871 contacts. The authorities took measures to isolate, transport, and monitor these contacts. Hainan provincial digital contact tracing identified 378 individuals with the highest infection risk (that is, the highest numbers of contacts and exposures), including 106 close contacts and 154 second-degree contacts who traveled on the same vehicles, 110 contacts within the same communities, and 8 contacts within the same malls. The Hainan Provincial Big Data Administration identified 6 high-risk communities and a number of high-risk shopping malls. According to the list of the highest-risk infected individuals and high-risk shopping malls and communities, the Command of Hainan Provincial COVID-19 Epidemic Prevention and Control directed health committees, the CDC, medical and health institutions, grassroot governments, and community residents’ committees to take mandatory measures (such as nucleic acid testing, isolation, and intensive treatment) for the highest-risk infected individuals and to implement emergency shutdown measures for high-risk public places. As a result, a patient who was not detected by traditional contact tracing was discovered through digital contact tracing, which provided vital information support to comprehensively curb the spread of the COVID-19 epidemic from the source.

## Discussion

### Main Findings

We summarized the organizational process, technical process, and main achievements of the graph database algorithm in tracing the contacts of COVID-19 cases in Hainan Province, China. This approach has practical importance in overcoming the limitations of traditional emergency management measures and existing digital contact tracing methods. From the perspective of the organizational process, our research found that Hainan Province formed a scientific and effective organizational structure and operating mechanism for digital contact tracing during the COVID-19 epidemic. The success of the organizational process is attributed to (1) the establishment of a special high-level administrative leadership agency to coordinate the entire process, namely, the Hainan Provincial Command of COVID-19 Epidemic Prevention and Control; (2) the clarification of the rights and obligations of relevant emergency management agencies in the digital contact tracing of the COVID-19 epidemic; (3) the formation of a flat and integrated organizational structure and operating mechanism for multiple agencies to communicate effectively in a timely manner and achieve collaborative governance; (4) the establishment of a dedicated big data management department to provide technical support required for data lifecycle management; and (5) the establishment of a government big data public service platform that supports the storage, recall, and analysis of multisource data according to the centralized model, and the application of the platform in digital contact tracing during the COVID-19 epidemic.

From the perspective of the technical process, our research is based on the use of the Neo4j graph database algorithm and ECharts data visualization tool to mine and analyze multisource COVID-19 epidemic big data. Hidden contacts and public places associated with confirmed patients, suspected patients, and asymptomatic infections were discovered and traced so that contacts and high-risk public places could be accurately identified. The results show that the digital contact tracing in Hainan Province is compatible with multisource heterogeneous epidemic big data; it can quickly and accurately find close, second-degree, or third-degree contacts, and it can identify public places with high risk of infection. Visualization technology is of great importance in optimizing public decision-making [27]. In our research, the interactive operating system for data visualization developed based on ECharts helps COVID-19 epidemic emergency management officials to simply and directly query populations and public places with high risk of infection, and it can dynamically monitor the epidemic situation to provide decision support for evidence-based emergency management strategies for the COVID-19 epidemic. The digital contact tracing system in Hainan Province can efficiently detect a large number of people and accurately locate the contacts of individuals with COVID-19, which overcomes the inefficiency and low completeness of traditional manual contact tracing and helps comprehensively curb the spread of the COVID-19 epidemic from the source.

### Application and Practice Prospects

Our research is expected to overcome the main difficulties faced in emergency management of the COVID-19 epidemic in different countries and regions due to their unique political, cultural, and civic concepts [18,19,28-31]. Our case study in Hainan Province found that digital contact tracing based on a graph database algorithm has high flexibility and agility in responding to the COVID-19 epidemic and may have greater application prospects in the aforementioned countries and regions. The majority of countries and regions, especially countries with more liberal policies, require more rapid and accurate strategies to identify and trace populations and public places with high risk of infection to adopt precise quarantine and emergency measures for these people and places instead of adopting mandatory measures for all citizens. Because of their agility and responsiveness, collaboration, communication, and evidence-based digital contact tracing have important applications in the early, middle, and late stages of the COVID-19 epidemic and in the small-scale rebound after confirmed cases are cleared. In short, our research results have broad application prospects for different countries and regions and in different stages of the COVID-19 epidemic.

### Limitations and Improvements

The main limitation of our research is the small coverage and insufficient information of COVID-19 epidemic data. Thus, tracing high-risk infected populations among the entire population of Hainan Province and their contacts is impossible. The participants in digital contact tracing in Hainan Province are limited to people with a history of residence in Hubei Province. This selection greatly reduces the scope of digital contact tracing. In addition, WeChat positioning–based GPS data, UnionPay consumption data, and railway and flight passenger information, which can accurately reflect the trajectory of residents, are owned by enterprises. These enterprises did not agree to provide users’ spatiotemporal trajectory data to the Hainan Provincial Big Data Administration to protect the privacy of their users.

Hainan Province needs to further improve the accuracy of data contact tracing using a graph database algorithm. Authorities need to adopt more comprehensive, accurate, and dynamic population spatiotemporal trajectory data to combat the epidemic. These data are the key to improve the accuracy of digital-based tracing. In addition, when confirmed, suspected, and asymptomatic infected individuals come into contact with the general population, the distance and duration of exposure are the main indicators for assessing the risk of cross-infection [10]. Hainan Province treats people who appeared in the same mode of transportation, shopping malls, or communities with confirmed, suspected, or asymptomatic infected individuals at the same time as contacts. This consideration lacks a threshold for exposure distance and duration and has a negative impact on the accuracy of contact tracing. Therefore, the digital contact tracing based on the graph database algorithm in Hainan Province needs to determine the thresholds of exposure distance and duration in different scenarios based on the professional opinions of experts in disease prevention and control and of medical staff. Digital technicians need to optimize the algorithm to improve the precision of digital contact tracing based on the thresholds of exposure distance and duration.

Furthermore, security issues in the process of digital contact tracing of epidemics based on a digital government platform under the centralized model need to be properly resolved in two aspects: governance and technology. In terms of governance, the law, system, and data management structure must be improved for the government to obtain citizens’ personal information in public health emergencies. Data sharing standards, authority management, and data security management in the process of epidemic data analysis and application, as the bases for the government to adopt useful data to respond to epidemics under the premise of ensuring the privacy of citizens, should be clarified. In addition, the government’s use of data from enterprises, social organizations, and other nongovernmental organizations to respond to the epidemic needs to be further clarified and improved. In terms of technology, data security technologies, such as cryptography methods, data encryption technologies, system vulnerability monitoring and repair technologies, virus-killing technologies, and automatic data deletion technologies, must be comprehensively used in the digital platform for epidemic emergency management.

Finally, the methods of collecting epidemic data were not sufficiently intelligent. For example, epidemiological investigation data were mainly collected by health personnel through face-to-face interviews with the cooperation of community resident committees. These data were manually entered into the information system by the staff of the CDC or medical institutions. This data collection method was relatively inefficient and requires substantial staffing. Furthermore, onsite investigation by staff increases the risk of cross-infection. Therefore, for digital contact tracing, it is necessary to develop more intelligent means of completion. Therefore, authorities should follow the development trend of governance in the digital age; develop an epidemic emergency management big data platform with the aid of governmental big data platforms; and fully use cloud transmission, cloud storage, and cloud computing technology to realize the real-time and dynamic collection of epidemic data from the transaction information systems of public and private agencies through a network interface to replace the traditional methods of collecting multisource epidemic data.

### Conclusion

Hainan Province, China, was selected as the case for this study. The use of a graph database algorithm and ECharts visualization tools in Hainan Province to trace contacts and identify high-risk public places was summarized in a centralized model during the process of emergency management of the COVID-19 epidemic. Moreover, the organizational and technical processes of the case we studied can help government agencies to implement precise emergency management measures and provide agile, evidence-based decision support to improve the responsiveness of the public health emergency response system. The organizational arrangements and technology involved in our research can be applied in different countries and regions to respond to the COVID-19 epidemic and can be applied at different stages of the COVID-19 epidemic. Future research should focus on solving the problems of insufficient data coverage information, insufficient contact tracing accuracy, insufficient intelligence of data collection methods, security concerns, and insufficient real-time performance of data sharing caused by the tradeoff between citizen privacy and public health security to optimize digital contact tracing.

## Abbreviations

QR: quick response
Wi-Fi: Wireless Fidelity
CDC: Center for Disease Control and Prevention
